# Associations Between Movement Behaviors and Emotional Changes in Toddlers and Preschoolers During Early Stages of the COVID-19 Pandemic in Chile

**DOI:** 10.3389/fped.2021.667362

**Published:** 2021-08-31

**Authors:** Nicolas Aguilar-Farias, Marcelo Toledo-Vargas, Sebastian Miranda-Marquez, Andrea Cortinez-O'Ryan, Pia Martino-Fuentealba, Carlos Cristi-Montero, Fernando Rodriguez-Rodriguez, Paula Guarda-Saavedra, Borja Del Pozo Cruz, Anthony D. Okely

**Affiliations:** ^1^Department of Physical Education, Sports and Recreation, Universidad de La Frontera, Temuco, Chile; ^2^UFRO Activate Research Group, Universidad de La Frontera, Temuco, Chile; ^3^IRyS Group, Physical Education School, Pontificia Universidad Católica de Valparaíso, Valparaíso, Chile; ^4^Centre for Active and Healthy Ageing, Department of Sports Science and Clinical Biomechanics, University of Southern Denmark, Odense, Denmark; ^5^School of Health and Society and Early Start, Faculty of Arts, Social Sciences and Humanities, Australia and Illawarra Health and Medical Research Institute, University of Wollongong, Wollongong, NSW, Australia

**Keywords:** physical activity, screen time, sleeping, emotion, coronavirus, stress, children

## Abstract

**Background:** There is limited evidence about emotional and behavioral responses in toddlers and preschoolers during the novel coronavirus (COVID-19) pandemic, particularly in Latin America.

**Objective:** To assess associations between changes in movement behaviors (physical activity, screen time and sleeping) and emotional changes in toddlers and preschoolers during early stages of the pandemic in Chile.

**Methods:** A cross-sectional study conducted from March 30th to April 27th, 2020. Main caregivers of 1- to 5-year-old children living in Chile answered an online survey that included questions about sociodemographic characteristics, changes in the child's emotions and behaviors, movement behaviors and caregivers' stress during the pandemic. Multiple linear regressions were used to assess the association between different factors and emotional changes in toddlers and preschoolers.

**Results:** In total, 1727 caregivers provided complete data on emotional changes for children aged 2.9 ± 1.36 years old, 47.9% girls. A large proportion of toddlers and preschoolers in Chile experienced emotional and behavioral changes. Most caregivers reported that children “were more affectionate” (78.9%), “more restless” (65.1%), and ‘more frustrated' (54.1%) compared with pre-pandemic times. Apart from changes in movement behaviors, factors such as child age, caregivers' age and stress, and residential area (urban/rural) were consistently associated with changes in emotions and behaviors.

**Conclusion:** The pandemic substantially affected the emotions and behaviors of toddlers and preschoolers in Chile. The findings suggest that supportive actions for caregivers may have a positive impact not only on adults but also on children. Mental health promotion programs should consider multilevel approaches in which the promotion of movement behaviors and support for caregivers should be essential pieces for future responses.

## Introduction

The coronavirus (COVID-19) pandemic declared by the World Health Organization (WHO) in March 2020, has affected millions of people worldwide (1). Latin America has been impacted significant in terms of mortality and the spread of the disease especially during the first wave. Chile accumulated 13,944 deaths from March until October 27^th^ 2020, placing the country seventh in the world with 74.5 deaths per 100,000 inhabitants due to COVID-19 (2).

One of the most common measures to control the spread of the virus was the introduction of mobility restrictions (i.e., lockdown) and physical distancing in the population. Despite their relevance, these measures affected a wide range of everyday life dimensions such as work, education, transport, recreation and household activities (1, 3). A large proportion of adults faced financial struggles due to job losses or suspensions, while children and adolescents experienced changes in their education, limits on their social interactions, and less access to school-based social services beyond education, such as food and health care (3, 4). These multiple challenges have likely had a negative impact not only on people's physical health but also on their psychosocial well-being (5).

Stress and social isolation are factors that affect mental and brain health (6), conditions that may be aggravated in families suffering health and economic hardships during the pandemic. Studies have shown increases in the rates of stress, depression and anxiety in adults during the COVID-19 pandemic (7–10). Children, particularly those under the age of five (11, 12) have been among the most vulnerable groups due to their reliance on others (parental, family, peer or institutional support) to cope with these challenging circumstances (7, 13, 14). During the initial stages of the pandemic, in Spain and Italy, most parents (87.5%) reported emotional and behavioral changes in children, and three out of four parents reported feeling stressed (15). In addition, children experienced more struggles in concentrating (76.6%), and were more irritable (39.0%), restless or nervous (38.0%), manifesting symptoms of loneliness (31.1%) (15). In China, children aged 3-6 were more likely to show clinginess and fear that family members may be sick due to COVID-19 compared with older children (16).

Mental health symptoms usually appear in childhood and then continue into adolescence (17–19). It may be expected to observe emotional and behavioral changes as a reaction of an adverse event like the pandemic, but these changes may also be affected by the caregivers' responses (20–23). Studies have shown that toddlers' and preschoolers' behavioral problems and hyperactivity were associated with their parents' mental health (24). Longitudinal evidence has shown that “proximal risks” such as family grief/illness events, harsh discipline, maternal emotional distress, overinvolved/protective parenting, have the most considerable effect on externalizing and internalizing symptoms of mental health in preschoolers (20). These potential risk factors may be exacerbated during the COVID-19 pandemic as most families are suffering a contextual change that has transformed family dynamics. However, more than 12 months after the declaration of the COVID-19 pandemic, limited evidence exists regarding the emotional impact on toddlers and preschoolers and how this is associated with their parents' feelings or behavioral responses.

Young children's movement behaviors (physical activity, sedentary behavior and sleep) have been negatively impacted during the pandemic (25, 26). This in turn has the potential of affecting other areas of health and development (27, 28). For example, physical activity is positively associated with psychosocial health (29) and with sociability (30). High levels of sedentary screen time during infancy has been shown to be associated with greater emotional and behavioral problems at age 4 (31) while preschoolers who met the WHO guideline for screen time had fewer emotional problems than preschoolers who did not meet the guideline (32). Shorter sleep duration has also been associated with poorer emotional regulation (33). This study aimed to evaluate the associations between changes in movement behaviors, caregivers' stress, and sociodemographic factors with emotional and behavioral responses in toddlers and preschoolers during early stages of the pandemic in Chile.

## Materials and Methods

### Participants and Procedures

An online survey for main caregivers of 1- to 5-year-old children living in Chile was conducted from March 30th to April 27th, 2020. The study was promoted online using social networks (Facebook, Twitter, and Instagram), messaging apps and emails to educational institutions that targeted all regions in Chile. The inclusion criteria were: (1) living in Chile, (2) being the main caregiver of a 1- to 5-year-old child, and (3) living with the child most of the time before and during the COVID-19 pandemic. The current study presents the results derived from a second survey completed by caregivers who participated in a study which aimed to assess movement behavior changes during the pandemic. All participants gave their online informed consent to participate in the study. The study was approved by the Scientific Ethics Committee at Universidad de La Frontera, Chile (ORD.: 009-2020).

The study started 2 weeks after the Chilean government mandated that educational centers close (March 16th, 2020) due to COVID-19 and finished on April 27^th^, 2020, when educational centers were still closed. Data were collected and managed using REDCap (Research Electronic Data Capture) hosted at the Universidad de La Frontera (34).

### Outcome

The emotional changes during the early stages of the pandemic were measured with questions developed for the context in COVID-19 pandemic. The emotions included in the study were selected from those commonly reported in the literature and used in questionnaires such as the Revised Children's Anxiety and Depression Scale (35) and the Strengths and Difficulties Questionnaire (36). The main caregivers answered the following question for ten emotions or attitudes: “During the last time in the context of the coronavirus pandemic (lockdown or isolation) the child has been/had more: affectionate/ restless/ aggressive/ irritable/ temper tantrums/ frustrated/ worried/ sad/ sensitive/ afraid?”. Each question had a Likert-type response options in a 5-point scale (Strongly disagree to Strongly agree), with an additional option for “not applicable”. The questionnaires were piloted in a small sample of caregivers before its official launch to assess pertinence, readability and understanding of the items. The questions related to emotional changes had a good internal consistency (Cronbach's alpha: 0.88). The questionnaire is included in the Supplementary Material.

### Movement Behaviors

Caregivers were asked to estimate total physical activity, screen time, sleep duration on a typical day before and during early stages of the COVID-19 pandemic. Sleep quality both before and during the COVID-19 pandemic was asked using a scale from 1 to 7 in which a higher score indicated better quality. These questions were adapted from those used in the International Study of Movement Behaviors in the Early Years (SUNRISE study, www.sunrise-study.com) to capture the unique features of the pandemic (37, 38). The changes in these behaviors were calculated using a residualized change score approach to eliminate auto-correlated errors and regression toward the mean (39, 40). Thus, first, each behavior during the COVID-19 pandemic was regressed on the behavior before the COVID-19. Then, the residualized change score (i.e., trend) for each behavior was estimated as each participant's standardized residual score. A positive residualized change score indicates an increase in the specific behavior from the time before COVID-19 and a negative score indicates a decrease.

### Covariates

Sociodemographic information included child's and caregiver's sex, child's and caregiver's age, caregiver's change in working condition due to the pandemic (yes/no, being fired or salary decreased), caregiver's stress during the pandemic (more irritable, more tired, having difficulties to concentrate, having difficulties to work; scale 1 to 5 [never to always]). The questions related to caregivers' stress had acceptable internal consistency (Cronbach's alpha: 0.79). The items are included in the Supplementary Material. Family characteristics included family income (<530 United States Dollars [USD]; ≥530– <1830 USD, ≥1830 USD), main caregiver's level of education, number of people per home, and number of children per home. Home characteristics included dwelling type (house, apartment or other), squared meters per person at home (<11.7 m^2^ per person, ≥11.7 to <18.3 m^2^ per person, ≥18.3 to <25 m^2^ per person, and ≥25 m^2^ per person), space to play at home (yes/no), living area (urban/rural) and living in an area under lockdown (yes/no).

### Statistical Analysis

Mean (standard deviation, SD) and proportions were used to describe the participants' characteristics. Comparisons between participants' characteristics and outcomes by sex were performed using *t*-tests and chi-squared test. Multiple linear regressions were used to assess the association between different factors and emotional changes in toddlers and preschoolers during early stages of the COVID-19 pandemic. All models were mutually adjusted for individual, caregivers, family, home and geographic characteristics describe above. All data were analyzed using Stata 15.1 (StataCorp LLC, USA). *P*-values < 0.05 were considered statistically significant (tested 2-sided).

## Results

In total, 1727 caregivers provided complete data on the emotional changes in their children. The mean age was 2.9 ± 1.36 years, and comprised 47.9% girls, corresponding to a 54.7% of those who completed the first stage of this study that included questions on movement behaviors (*n* = 3,157). No differences were observed in the sample characteristics with the original sample. Children reduced their physical activity, increased their screen time and their sleep quality declined during the early stages of the pandemic. About 60% of caregivers were 25- to 34-years-old and about 40% experienced changes in their working conditions. On a scale from 1 to 5, with a higher score indicating a worse outcome, caregivers scored 3.4 ± 1.06 for being more irritable, 3.7 ± 1.12 for being more tired, 3.5 ± 1.18 for having difficulties in concentrating, and 3.4 (1.41) for having difficulties with work. Family characteristics were comparable with those observed in the National Census for the corresponding age group in terms of dwelling (80.6 vs. 79.7% living in a house) and living area (10.8 vs. 13.5% living in a rural area); however, the current sample was more educated (68.1 vs. 39.7% with more than 12 years of education) (41). About 80% of participants were in lockdown when the questionnaire was completed. Further details regarding the sample are shown in Table 1.

**Table 1:** Sample characteristics.

**Characteristic**	**Total** **(***n*** = 1,727)**	**Boys** **(***n*** = 900)**	**Girls** **(***n*** = 827)**	***p*** ^**a**^
**Individual characteristics**				
Age, mean years (SD)	2.9 (1.37)	2.9 (1.36)	3.0 (1.38)	0.114
**Overall change, mean (SD)**				
Physical activity (h/day)	−0.80 (1.65)	−0.78 (1.63)	−0.82 (1.67)	0.609
Screen time (h/day)	1.44 (1.55)	1.48 (1.60)	1.40 (1.48)	0.282
Sleep duration (h/day)	0.03 (1.62)	0.02 (1.64)	0.04 (1.60)	0.764
Sleeping quality (score 1 to 7)	−0.78 (1.64)	−0.81 (1.60)	−0.74 (1.69)	0.417
Enrolled in ECEC, yes, n (%)	1342 (77.7)	700 (77.8)	642 (77.6)	0.941
**Main caregiver's characteristics**				
Sex, female, *n* (%)	1,668 (96.6)	858 (95.3)	810 (97.9)	0.003
**Age category**, ***n*****(%)**				
<25 y	205 (11.9)	113 (12.6)	92 (11.1)	0.700
25 to <35 y	1009 (58.4)	523 (58.1)	486 (58.8)	
35 to <45 y	486 (28.1)	252 (28.0)	234 (28.3)	
45 y and more	27 (1.6)	12 (1.3)	15 (1.8)	
**Main caregiver's level of education**, ***n*****(%)**				
Incomplete high school	37 (2.1)	18 (2.0)	19 (2.3)	0.943
Complete high school	514 (29.8)	270 (30.0)	244 (29.5)	
Technical degree	210 (12.2)	112 (12.4)	98 (11.9)	
University degree	966 (55.9)	500 (55.6)	466 (56.4)	
Work changes due to pandemic, yes, *n* (%)	642 (37.2)	344 (38.2)	298 (36.0)	0.347
**Stress symptoms, mean (SD), 1 to 5 scale**				
More irritable	3.4 (1.06)	3.4 (1.07)	3.4 (1.05)	0.241
More tired	3.7 (1.12)	3.8 (1.09)	3.7 (1.14)	0.070
Having difficulties to concentrate	3.5 (1.18)	3.6 (1.16)	3.5 (1.18)	0.022
Having difficulties to work	3.4 (1.41)	3.4 (1.38)	3.3 (1.44)	0.011
**Family characteristics**				
**Family income**, ***n*****(%)**				
<530 USD	526 (30.5)	264 (29.3)	262 (31.7)	0.464
≥530– <1,830 USD	878 (50.9)	460 (51.1)	418 (50.5)	
≥1,830 USD	323 (18.7)	176 (19.6)	147 (17.8)	
**Number of people per home**, ***n*****(%)**				
3 or less	672 (38.9)	355 (39.4)	317 (38.3)	0.540
4	534 (30.9)	284 (31.6)	250 (30.2)	
5 or more	521 (30.2)	261 (29.0)	261 (29.0)	
**Children per home**, ***n*****(%)**				
1 child	861 (49.8)	457 (50.8)	404 (48.9)	0.022
2 children	633 (36.7)	341 (37.9)	292 (35.3)	
3 or more	233 (13.5)	102 (11.3)	131 (15.9)	
**Home characteristics**				
**Dwelling type**, ***n*****(%)**				
House	1,392 (80.6)	738 (82.0)	654 (79.1)	0.286
Apartment	283 (16.4)	138 (15.3)	145 (17.6)	
Other	52 (3.0)	24 (2.7)	28 (3.4)	
**Squared meters per person at home**, ***n*****(%)**				
<11.7 m^2^ per person	415 (24.0)	208 (23.1)	207 (25.0)	0.749
≥11.7 to <18.3 m^2^ per person	398 (23.1)	206 (22.9)	192 (23.2)	
≥18.3 to <25 m^2^ per person	415 (24.0)	218 (24.2)	197 (23.8)	
≥25 m^2^ per person	499 (28.9)	268 (29.8)	231 (27.9)	
Space to play at home, yes, *n* (%)	1,604 (92.9)	835 (92.8)	769 (93.0)	0.866
**Geographical characteristics**				
Living area, urban, *n* (%)	1,541 (89.2)	799 (88.8)	742 (89.7)	0.527
Lockdown, yes, *n* (%)	1,356 (78.5)	703 (78.1)	653 (79.0)	0.668

a *P-value for the differences between boys and girls for each variable. USD, United States Dollars; SD, standard deviation*.

### Emotional Changes in Toddlers and Preschoolers

**Figure 1:**
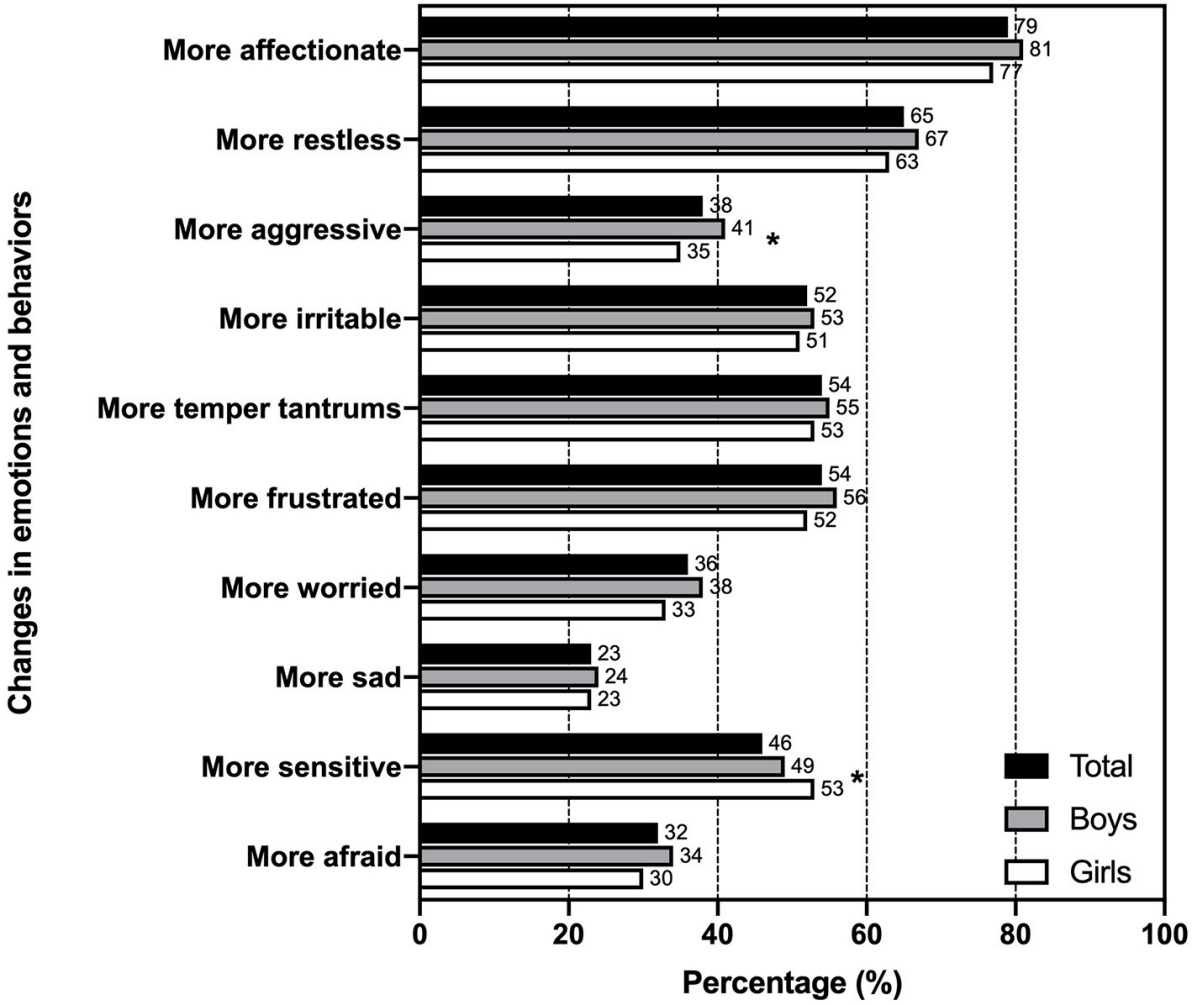
Proportion of children that showed emotional and behavioral changes during early stages of the COVID-19 pandemic in Chile. Emotional changes were measured with a 1 to 5 scale in which 4 or more indicated change. Parents were allowed to answer “not applicable.” *Indicates difference between boys and girls (*p* < 0.05).

Figure 1 shows the proportion of children whose parents reported emotional changes during early stages of the pandemic in Chile. Most caregivers reported that children were “more affectionate” (78.9%), “more restless” (65.1%), and “more frustrated” (54.1%) compared with pre-pandemic times. The least frequently reported emotional changes were “more sad” (23.4%), “more worried” (35.5) and “more afraid” (31.9%). The only differences according to sex were observed for “more aggressive” (40.5% in boys vs. 35.2% in girls, *p* = 0.032) and “more sensitive” (48.5% in boys vs. 52.6% in girls, *p* = 0.024).

### Factors Associated With Emotional Changes in Toddlers and Preschoolers

Table 2 shows the results from the multivariable linear regression models in which different factors were associated with emotional changes in children during early stages of the COVID-19 pandemic in Chile. At the individual level, being a boy was associated with increases in aggressiveness and irritability. Older children were less likely to show increases in restlessness, irritability, temper tantrums, but at the same time they were more worried, sad and afraid than younger children.

**Table 2:** Child's and caregivers'characteristics associated with emotional changes in toddlers and preschoolers during early stages of the COVID-19 pandemic (*n* = 1,727).

**Characteristic**	**Affectionate ß (95%CI)**	**Restless ß (95%CI)**	**Aggressive ß (95%CI)**	**Irritable ß (95%CI)**	**Temper tantrums ß (95%CI)**	**Frustrated ß (95%CI)**	**Worried ß (95%CI)**	**Sad ß** ** (95%CI)**	**Sensitive ß (95%CI)**	**Afraid ß (95%CI)**
**Child's characteristics**										
**Sex (ref: girls)**										
Boys	0.02 (−0.08, 0.13)	0.10 (−0.02, 0.22)	0.24^***^ **(0.10, 0.37)**	0.15^*^ **(0.03, 0.28)**	0.08 (−0.05, 0.20)	0.09 (−0.03, 0.22)	0.11 (−0.01, 0.24)	0.04 (−0.08, 0.17)	0.08 (−0.05, 0.21)	0.08 (−0.05, 0.22)
Age, years	0.03 (−0.01, 0.07)	–0.08^***^ **(–**0.13**, –0.03)**	−0.03 (−0.08, 0.03)	–0.10^***^ **(–**0.15**, –0.06)**	–0.06^***^ **(–**0.11**, –0.01)**	−0.03 (−0.08, 0.02)	0.13^***^ **(0.08, 0.18)**	0.11^***^ **(0.06, 0.16)**	0.05 (0.00, 0.10)	0.07^**^ **(0.02, 0.12)**
**Change score in movement behaviors** ^**a**^										
Physical activity	0.07^*^ **(0.01, 0.13)**	0.06 (−0.01, 0.12)	−0.01 (−0.09, 0.06)	–0.08^*^ **(–**0.15**, –0.01)**	−0.04 (−0.12, 0.03)	−0.05 (−0.12, 0.02)	–0.10^**^ **(–**0.17**, –0.05)**	−0.06 (−0.13, 0.01)	–0.07^*^ **(–0.15, 0.00)**	–0.12^**^ **(–**0.20**, –0.04)**
Screen time	0.01 (−0.04, 0.07)	0.06 (0.00, 0.13)	0.12^**^ **(0.04, 0.19)**	0.12^***^ **(0.06, 0.19)**	0.10^**^ **(0.03, 0.17)**	0.13^***^ **(0.06, 0.19)**	0.05 (−0.01, 0.12)	0.06 (−0.01, 0.13)	0.08^*^ **(0.01, 0.15)**	0.01 (−0.07, 0.08)
Sleep duration	0.07^**^ **(0.02, 0.12)**	−0.04 (−0.10, 0.02)	–0.08^*^ **(–**0.15**, –0.02)**	−0.03 (−0.09, 0.03)	–0.06^*^ **(–**0.13**, –0.00)**	−0.02 (−0.08, 0.04)	−0.04 (−0.10, 0.02)	–0.09^**^ **(–**0.15**, –0.03)**	−0.06 (−0.13, 0.00)	−0.02 (−0.08, 0.05)
Sleep quality	0.04 (−0.01, 0.10)	–0.19^***^ **(–**0.25**, –0.12)**	–0.20^***^ **(–**0.27**, –0.13)**	–0.29^***^ **(–**0.36**, –0.22)**	–0.26^***^ **(–**0.32**, –0.19)**	–0.27^***^ **(–**0.33**, –0.20)**	–0.22^***^ **(–**0.29**, –0.16)**	–0.20^***^ **(–**0.27**, –0.13)**	–0.20^***^ **(–**0.27**, –0.13)**	–0.24^***^ **(–**0.31**, –0.17)**
Enrolled in ECEC (ref: no)	0.08 (−0.06, 0.22)	0.12 (−0.04, 0.28)	0.02 (−0.16, 0.20)	0.04 (−0.13, 0.21)	0.11 (−0.06, 0.29)	0.17 (−0.00, 0.33)	0.12 (−0.05, 0.29)	0.01 (−0.16, 0.19)	0.07 (−0.11, 0.25)	0.05 (−0.14, 0.23)
**Caregiver's characteristics**										
**Age category (ref: <25 y)**										
25 to <35 years	0.00 (−0.17, 0.18)	−0.04 (−0.24, 0.16)	−0.19 (−0.42, 0.04)	−0.20 (−0.41, 0.02)	−0.19 (−0.41, 0.03)	−0.19 (−0.40, 0.02)	−0.09 (−0.30, 0.13)	0.04 (−0.18, 0.26)	−0.15 (−0.38, 0.08)	0.06 (−0.18, 0.29)
35 to <45 years	−0.15 (−0.36, 0.05)	−0.09 (−0.32, 0.15)	−0.13 (−0.40, 0.14)	−0.22 (−0.47, 0.03)	−0.18 (−0.43, 0.08)	–0.27^*^ **(–**0.52**, –0.02)**	−0.00 (−0.25, 0.25)	0.12 (−0.13, 0.37)	−0.22 (−0.48, 0.04)	0.10 (−0.18, 0.37)
45 years and more	−0.38 (−0.83, 0.07)	–0.59^*^ **(–**1.11**, –0.08)**	–0.60^*^ **(–**1.17**, –0.03)**	–0.57^*^ **(–**1.12**, –0.02)**	–0.71^*^ **(–**1.27**, –0.15)**	–0.71^**^ **(–**1.24**, –0.18)**	−0.10 (−0.63, 0.43)	−0.10 (−0.64, 0.43)	−0.29 (−0.87, 0.28)	−0.23 (−0.83, 0.38)
**Level of education (ref: incomplete high school)**										
Complete high school	0.03 (−0.34, 0.39)	−0.07 (−0.48, 0.35)	0.01 (−0.46, 0.48)	0.34 (−0.10, 0.79)	−0.02 (−0.47, 0.44)	0.20 (−0.24, 0.63)	0.16 (−0.27, 0.59)	−0.16 (−0.62, 0.29)	−0.01 (−0.46, 0.43)	−0.25 (−0.73, 0.22)
Technical degree	0.08 (−0.30, 0.47)	−0.18 (−0.62, 0.26)	0.03 (−0.47, 0.52)	0.25 (−0.22, 073)	−0.03 (−0.51, 0.45)	0.26 (−0.20, 0.71)	0.16 (−0.29, 0.61)	−0.26 (−0.74, 0.21)	0.06 (−0.41, 0.53)	−0.33 (−0.83, 0.17)
University degree	0.06 (−0.31, 0.43)	−0.12 (−0.54, 0.31)	0.09 (−0.39, 0.57)	0.33 (−0.13, 0.79)	−0.01 (−0.48, 0.45)	0.31 (−0.13, 0.75)	0.19 (−0.25, 0.63)	−0.27 (−0.73, 0.20)	−0.07 (−0.53, 0.38)	−0.33 (−0.82, 0.15)
**Work change (ref: no)**										
Yes	−0.04 (−0.15, 0.07)	−0.09 (−0.22, 0.04)	0.00 (−0.15, 0.14)	−0.03 (−0.17, 0.10)	−0.03 (−0.17, 0.12)	0.04 (−0.10, 0.17)	0.04 (−0.09, 0.17)	0.03 (−0.11, 0.17)	0.06 (−0.08, 0.21)	0.06 (−0.09, 0.20)
**Stress symptoms (1 to 5 scale)**										
Irritable	0.01 (−0.05, 0.07)	0.24^***^ **(0.17, 0.31)**	0.26^***^ **(0.18, 0.34)**	0.29^***^ **(0.21, 0.36)**	0.25^***^ **(0.17, 0.33)**	0.24^***^ **(0.16, 0.31)**	0.16^***^ **(0.08, 0.24)**	0.16^***^ **(0.08, 0.24)**	0.21^***^ **(0.13, 0.29)**	0.19^***^ **(0.11, 0.28)**
Tired	−0.01 (−0.08, 0.05)	0.04 (−0.03, 0.11)	0.10^*^ **(0.02, 0.18)**	0.08 (0.00, 0.15)	0.07 (−0.01, 0.15)	0.09^*^ **(0.01, 0.17)**	0.05 (−0.03, 0.13)	0.02 (−0.07, 0.10)	−0.05 (−0.14, 0.03)	−0.02 (−0.11, 0.06)
Difficulties to concentrate	0.07^*^ **(0.01, 0.13)**	0.06 (−0.01, 0.13)	0.02 (−0.06, 0.10)	0.04 (−0.04, 0.12)	0.04 (−0.04, 0.12)	0.06 (−0.02, 0.13)	0.04 (−0.03, 0.12)	0.08^*^ **(0.00, 0.16)**	0.18^***^ **(0.10, 0.26)**	0.07 (−0.01, 0.16)
Difficulties to work	0.00 (−0.05, 0.04)	−0.01 (−0.06, 0.04)	0.04 (−0.02, 0.09)	0.00 (−0.05, 0.05)	0.01 (−0.04, 0.07)	0.00 (−0.06, 0.05)	0.01 (−0.04, 0.07)	0.02 (−0.04, 0.07)	0.03 (−0.03, 0.08)	0.05 (−0.01, 0.10)

a *standardized residualized change score between the behavior before and during the pandemic*.

*Emotions were in a scale from 1 to 5, participants had the chance to select not applicable*.

*Each model was adjusted by individual, caregiver's, family, home and geographical characteristics, including region*.

*Significance*:

*** * = p < 0.001*;

** * = p < 0.01*;

* * = p < 0.05*.

*Bold numbers indicate statistical significance*.

Children whose parents reported smaller declines in physical activity levels were more affectionate and less irritable, worried, sensitive and afraid (Table 2). Those who had greater increases in their screen time were more likely to be more aggressive, irritable, frustrated, sensitive and have more temper tantrums. Children whose sleep duration increased were more affectionate and less aggressive, angry and sad. Children whose sleep quality was less affected were less likely to be restless, aggressive, irritable, have temper tantrums, frustrated, worried, sad, sensitive and afraid.

Caregivers who were 45 years and older reported that their children were less likely to be more restless, aggressive, irritable and had fewer temper tantrums, while caregivers who were 35 years and older reported less frustration in their children (Table 2). Irritability from caregivers was positively associated with all the measured emotions except for affection. Tiredness from caregivers was positively associated with aggressiveness, frustration, sadness and sensitivity, and fear. Those caregivers who reported more difficulties in concentrating had children who were more affectionate, sad and sensitive.

When observing the family characteristics (Table 3), those children from wealthier families were less likely to be worried and sad. Children who lived with five or more people were more restless. In contrast, those who lived with three or more children were less likely to be restless. Children who lived in homes with between 18.3 and 25 m^2^ per person were less likely to be more aggressive. Those children who lived in rural areas were less restless, irritable, frustrated and sensitive, and had fewer temper tantrums. Children who were under lockdown measures were less likely to be sad and afraid.

**Table 3:** Family, home and geopolitical characteristics associated with emotional changes in toddlers and preschoolers during early stages of the COVID-19 pandemic (*n* = 1,727).

**Characteristic**	**Affectionate ß (95%CI)**	**Restless ß (95%CI)**	**Aggressive ß (95%CI)**	**Irritable ß (95%CI)**	**Temper tantrums ß (95%CI)**	**Frustrated ß (95%CI)**	**Worried ß (95%CI)**	**Sad ß** **(95%CI)**	**Sensitive ß (95%CI)**	**Afraid ß (95%CI)**
**Family characteristics**										
**Family income (ref: <530 USD)**										
≥530- <1,830 USD	0.02 (−0.11, 0.16)	−0.09 (−0.25, 0.07)	−0.08 (−0.26, 0.09)	−0.04 (−0.20, 0.13)	0.02 (−0.15, 0.19)	−0.11 (−0.28, 0.05)	−0.13 (−0.29, 0.03)	−0.12 (−0.29, 0.04)	−0.06 (−0.23, 0.12)	−0.03 (−0.21, 0.14)
≥1830 USD	−0.07 (−0.26, 0.12)	−0.19 (−0.41, 0.03)	−0.21 (−0.46, 0.04)	−0.19 (−0.42, 0.04)	0.04 (−0.20, 0.28)	−0.17 (−0.40, 0.06)	–0.26^*^ **(–**0.49**, –0.03)**	–0.35^**^ **(–**0.58**, –0.12)**	−0.20 (−0.44, 0.04)	−0.23 (−0.48, 0.01)
Number of people per home (ref: 3 or less)										
4	0.09 (−0.07, 0.26)	0.17 (−0.02, 0.36)	0.03 (−0.19, 0.24)	0.18 (−0.02, 0.38)	0.05 (−0.15, 0.26)	0.05 (−0.15, 0.25)	0.01 (−0.19, 0.21)	0.01 (−0.19, 0.21)	−0.07 (−0.28, 0.14)	0.04 (−0.17, 0.25)
5 or more	0.08 (−0.10, 0.25)	0.25^*^ **(0.05, 0.44)**	0.07 (−0.15, 0.30)	0.10 (−0.11, 0.31)	0.13 (−0.08, 0.35)	0.08 (−0.13, 0.29)	0.09 (−0.12, 0.29)	0.00 (−0.21, 0.21)	−0.11 (−0.33, 0.11)	0.05 (−0.17, 0.28)
Children per home (ref: 1 child)										
2 children	−0.04 (−0.19, 0.11)	−0.04 (−0.21, 0.13)	0.16 (−0.04, 0.35)	−0.03 (−0.21, 0.15)	0.03 (−0.16, 0.22)	0.06 (−0.12, 0.24)	0.15 (−0.03, 0.33)	0.17 (−0.01, 0.35)	0.07 (−0.12, 0.26)	0.02 (−0.17, 0.21)
3 or more	−0.05 (−0.25, 0.15)	–0.25^*^ **(–**0.48**, –0.01)**	0.04 (−0.23, 0.30)	0.05 (−0.20, 0.30)	−0.02 (−0.27, 0.23)	0.02 (−0.23, 0.27)	0.03 (−0.21, 0.28)	0.06 (−0.19, 0.31)	0.18 (−0.08, 0.44)	0.01 (−0.26, 0.27)
**Home characteristics**										
**Dwelling type (ref: house)**										
Apartment	0.13 (−0.02, 0.28)	0.11 (−0.06, 0.29)	−0.07 (−0.26, 0.13)	0.00 (−0.18, 0.18)	−0.03 (−0.22, 0.15)	0.03 (−0.15, 0.21)	0.01 (−0.17, 0.19)	0.08 (−0.11, 0.26)	0.08 (−0.11, 0.27)	−0.02 (−0.22, 0.17)
Other	0.09 (−0.22, 0.39)	0.14 (−0.22, 0.49)	0.29 (−0.12, 0.69)	0.26 (−0.11, 0.63)	0.19 (−0.18, 0.57)	0.30 (−0.07, 0.67)	0.01 (−0.35, 0.38)	0.05 (−0.33, 0.44)	0.08 (−0.31, 0.47)	0.33 (−0.06, 0.73)
**Squared meters per person at home (ref: <11.7 m^2^ per person)**										
≥11.7 to <18.3 m^2^ per person	0.02 (−0.13, 0.18)	−0.03 (−0.21, 0.14)	0.00 (−0.20, 0.20)	−0.13 (−0.32, 0.06)	−0.05 (−0.24, 0.14)	−0.06 (−0.25, 0.13)	−0.01 (−0.20, 0.18)	0.02 (−0.17, 0.21)	−0.09 (−0.29, 0.10)	0.05 (−0.15, 0.26)
≥18.3 to <25 m^2^ per person	0.09 (−0.08, 0.26)	0.09 (−0.11, 0.28)	0.24^*^ **(0.02, 0.46)**	0.16 (−0.04, 0.37)	0.21 (0.00, 0.41)	0.16 (−0.04, 0.37)	0.02 (−0.18, 0.22)	0.08 (−0.12, 0.29)	0.04 (−0.17, 0.26)	0.09 (−0.12, 0.31)
≥25 m^2^ per person	0.02 (−0.16, 0.20)	0.06 (−0.15, 0.26)	0.19 (−0.04, 0.43)	0.16 (−0.06, 0.38)	0.09 (−0.13, 0.31)	0.13 (−0.08, 0.35)	0.09 (−0.13, 0.30)	0.08 (−0.14, 0.30)	−0.05 (−0.28, 0.18)	0.09 (−0.15, 0.32)
**Space to play at home (ref: no)**										
Yes	0.19 (−0.02, 0.40)	−0.12 (−0.35, 0.12)	−0.02 (−0.28, 0.24)	−0.20 (−0.45, 0.05)	−0.29 (−0.54, −0.03)	−0.23 (−0.48, 0.01)	−0.04 (−0.28, 0.21)	0.02 (−0.23, 0.27)	−0.19 (−0.46, 0.07)	−0.09 (−0.35, 0.18)
**Geographical characteristics**										
**Area (ref: urban)**										
Rural	−0.09 (−0.26, 0.08)	–0.32^**^ **(–**0.52**, –0.13)**	−0.22 (−0.44, 0.00)	–0.23^*^ **(–**0.43**, –0.02)**	–0.23^**^ **(–**0.44**, –0.02)**	–0.28^*^ **(–**0.48**, –0.07)**	0.08 (−0.13, 0.28)	−0.07 (−0.28, 0.14)	–0.27^*^ **(–**0.48**, –0.05)**	−0.06 (−0.28, 0.16)
**Lockdown (ref: no)**										
Yes	0.00 (−0.12, 0.13)	−0.01 (−0.15, 0.14)	−0.11 (−0.28, 0.05)	−0.10 (−0.25, 0.06)	−0.12 (−0.27, 0.04)	−0.09 (−0.24, 0.06)	−0.11 (−0.26, 0.04)	–0.23^**^ **(–**0.39**, –0.08)**	−0.01 (−0.17, 0.15)	–0.24^**^ **(–**0.40**, –0.08)**

*Coefficients are standardized residualized change score between the behavior before and during the pandemic*.

*Emotions were in a scale from 1 to 5, participants had the chance to select not applicable*.

*Each model was adjusted by individual, caregiver's, family, home and geographical characteristics, including region*.

*Significance*:

** * = p < 0.01*;

* * = p < 0.05*.

*Bold numbers indicate statistical significance*.

## Discussion

To our knowledge, this is the first study in Latin America that has reported the impact of the pandemic on emotions and behaviors among toddlers and preschoolers. This study has shown that a large proportion of toddlers and preschoolers in Chile experienced emotional and behavioral changes during the early stages of the COVID-19 pandemic. Several variables were consistently associated with emotional changes such as the child's age, changes in movement behaviors in the child, caregivers' age, caregiver's irritability, and residential area. Some family and home characteristics such as family income and number of inhabitants per home were also associated with emotional changes but less consistently than the other factors. The presence of lockdown was inversely associated with children being more sad and afraid.

Our study showed that during the early stages of the pandemic child's and caregiver's characteristics were more consistently associated with emotional changes in toddlers and preschoolers than family, home and geographic characteristics. As shown in other studies (29–33), physical activity, screen time and sleep duration and quality were associated with emotions in children, highlighting the importance of ensuring opportunities to maintain healthy movement behaviors. These results capture early stages of the pandemic. Therefore, some of these associations may have changed as the restrictions to mobility mandated in Chile to control the spread of the virus were maintained for the remainder of 2020. The negative effect on healthy levels of movement behaviors and the stress associated with the restrictions may have negative effects not only on the emotional health but also cognitive development in children (42, 43). In line with international recommendations (27, 44), these findings suggest that healthy movement behaviors should be a key response to support psychosocial health in early childhood and prevent other deleterious effects of the pandemic.

Among the caregiver's characteristics, the study showed associations between caregivers' irritability, tiredness and difficulties in concentrating with changes in children's emotions and behaviors. Other studies have reported how parental distress has been associated with children's emotions and behaviors during the COVID-19 pandemic (45, 46). During the first months of the pandemic, our results show that changes in working conditions for the caregivers were not associated with emotional responses in children, but this may have changed as the COVID-19 pandemic progressed. Several family and home characteristics were associated with emotional changes in toddlers and preschoolers, highlighting the importance that emotional support from caregivers is to young children. These findings reinforce the need for implementing supportive strategies for caregivers as most of them reported increases in emotional stress, which is likely to be maintained or worsened as the pandemic continues (47). Programs should be supportive not only at individual level but also through comprehensive approaches in which communities, employers and decision-makers should understand and empathize with caregivers (mainly women in Chile). A model similar to that used in the “Sistema Distrital del Cuidado” (District system of care in English) in Bogotá (48), Colombia, that was developed prior to the COVID-19 pandemic, may be used as a starting point as it focused on providing opportunities for reducing the burden of care, particularly in women, while offering educational and health care activities, among others, for the users.

Living in rural areas was frequently associated with fewer emotional changes in children. During the COVID-19 pandemic, living in rural areas was also reported as a factor positively related to smaller decreases in physical activity, declines in sleep quality and increases in screen time (25). In this context, a study conducted in 14 countries showed that preschoolers who were able to go outside during the pandemic were more likely to meet the PA guidelines (26). To compensate for the lack of opportunity for some children who live in urban areas, strategies should provide opportunities to access green spaces or open spaces to play in cities while considering physical distancing due to the pandemic. This is particularly relevant in countries like Chile in which the opening of public and national parks has been postponed for months during the pandemic, whereas commercial areas have remained opened (49). As green spaces enhance well-being, overall health and cognitive development in children (50–53) and children who live further from green spaces are more sedentary and have poorer mental health (54), it is critical to find ways for children and families to access such spaces during the pandemic. Actions to promote healthy movement behaviors in urban areas such as open streets programs should be implemented to mitigate the lack of access to green and open spaces observed in most places not only during the pandemic but also in potential post-pandemic times (55, 56).

We acknowledge that more factors may contribute to the emotional and behavioral changes during the pandemic. Strategies to mitigate the negative socioemotional issues derived from the pandemic should include multilevel approaches for promoting more physical activity, less screen time and more and better sleep quality (25, 57). Policies must consider toddlers and preschoolers in their design. The length of the pandemic in terms of the age of the child is likely to have a considerable impact on their future development compared with adults (43, 57, 58). More effort is required to manage the collateral effects of the pandemic on mental health. A report from WHO showed that a large proportion of member states have mental health and psychological support plans, but only about a fifth of them have secured additional funding for covering the activities (1). Considering the benefits of healthy levels of physical activity, sedentary behavior and sleep on socioemotional health, we recommend governments, institutions and professionals secure funding and implement supportive strategies for caregivers, early childhood education and care services, and town planners to facilitate healthier behaviors in toddlers and preschoolers.

### Strengths and Limitations

Our study has explored the emotional and behavioral changes in toddlers and preschoolers using a socio ecological perspective, including the main caregiver's distress. This is critical in a pandemic context in which supporting networks (educational community, childcare services, families, friends) are more limited and caring responsibilities rely on fewer individuals. Although this was a cross-sectional study, under the circumstances of a natural experiment, we have provided evidence on a topic that is not frequently explored in movement behaviors, which is mainly focused on physical health (59). However, there are limitations in our study. The cross-sectional design of the study limits its ability to draw conclusions about causality. Also, the self- and proxy-reports used in this study may have been affected by different sources of bias such as recall or social desirability. Our study may have recruited caregivers who would have been more concerned regarding their family's health, including emotions and movement behaviors, affecting the composition of our sample. Although we used commonly used and freely available social networks to recruit participants throughout the entire country, the final sample was not entirely representative. The sample was more educated than that observed in the census for the same age group, but it was comparable in terms of dwelling type and living area (41). We recruited a large sample, but unfortunately only 55% completed the questionnaire. However, the characteristics of the sample of the current study were not different from those who completed the section on movement behaviors (25). The lower participation can be explained by people being more reluctant to provide personal information through online methods compared with face-to-face methodologies. We used the best possible option to measure the variables included in the study as strict health and ethical restrictions were mandated during the early stages of the pandemic (60) limiting, for example, the use of accelerometers or other instruments that may have required contact with participants. To overcome some of these limitations, ongoing studies should explore longitudinal associations between different exposure variables and mental health outcomes.

## Conclusions

The study showed that a large of proportion of toddlers and preschoolers in Chile showed negative emotional and behavioral changes during the early stages of the COVID-19 pandemic. These changes were associated with factors such as the child's age, a decrease in physical activity and sleep duration, an increase in screen time and a decline in sleep quality. Caregivers' characteristics, including age and irritability, were also associated with child's emotional changes during the pandemic. Living in rural areas was associated with less marked changes in emotions and behaviors. Mental health promotion programs should consider comprehensive and multilevel approaches in which promoting healthy levels of movement behaviors should be an essential intervention strategy. The findings suggest that supportive actions for caregivers may have a positive impact not only on adults but also on children. Governments should highlight the importance of healthy movement behaviors in their messages and actions during and post-pandemic through strong campaigns and through supporting environmental changes to facilitate more physical activity, less screen time and more and better sleep in toddlers and preschoolers.

## Data Availability Statement

The anonymised and raw data supporting the conclusions of this article will be made available by the corresponding author, upon reasonable request.

## Ethics Statement

The studies involving human participants were reviewed and approved by Scientific Ethics Committee at Universidad de La Frontera, Chile (ORD.: 009-2020). The patients/participants provided their written informed consent to participate in this study.

## Author Contributions

NA-F, PM-F, MT-V, AC-O'R, SM-M, and BP: conceptualization. NA-F, PM-F, MT-V, SM-M, CC-M, FR-R, and AC-O'R: methodology and investigation. MT-V and NA-F: software and validation. NA-F: formal analysis, data curation, writing—original draft preparation, and visualization. NA-F and PM-F: resources, project administration, and funding acquisition. NA-F, PM-F, and AO: supervision. All authors writing—review and editing. All authors have read and agreed to the published version of the manuscript.

## Conflict of Interest

The authors declare that the research was conducted in the absence of any commercial or financial relationships that could be construed as a potential conflict of interest.

## Publisher's Note

All claims expressed in this article are solely those of the authors and do not necessarily represent those of their affiliated organizations, or those of the publisher, the editors and the reviewers. Any product that may be evaluated in this article, or claim that may be made by its manufacturer, is not guaranteed or endorsed by the publisher.

## References

[1] World Health Organization. The impact of COVID-19 on Mental, Neurological and Substance Use Services: Results of a Rapid Assessment. Geneva: WHO (2020).

[2] Johns Hopkins University & Medicine. Coronavirus Resource Center. Mortality trends USA2020. Available online at: https://coronavirus.jhu.edu/data/mortality (accessed April 1, 2020).

[3] Golberstein E Wen H Miller BF. Coronavirus Disease 2019. (COVID-19) and mental health for children and adolescents. JAMA Pediatr. (2020) 174:819–20. 10.1001/jamapediatrics.2020.1456 32286618

[4] Crawley E Loades M Feder G Logan S Redwood S Macleod J. Wider collateral damage to children in the UK because of the social distancing measures designed to reduce the impact of COVID-19 in adults. BMJ Paediatr Open. (2020) 4:e000701. 10.1136/bmjpo-2020-000701 32420459 PMC7223269

[5] The Lancet Infectious Diseases. The intersection of COVID-19 and mental health. Lancet Infect Dis. (2020) 20:1217. 10.1016/S1473-3099(20)30797-0 33038942 PMC7544473

[6] World Health Organization. Mental health: strengthening our response Geneva, Switzerland (2018). Available online at: https://www.who.int/news-room/fact-sheets/detail/mental-health-strengthening-our-response (accessed January 12, 2021).

[7] United For Global Mental Health. The Impact of COVID-19 on Global Mental Health. A Brief 2020. London: United For Global Mental Health (2020).

[8] Wang C Pan R Wan X Tan Y Xu L Ho CS. Immediate psychological responses and associated factors during the initial stage of the 2019 coronavirus disease (COVID-19) epidemic among the general population in China. Int J Environ Res Public Health. (2020) 17:1729. 10.3390/ijerph17051729 32155789 PMC7084952

[9] Holmes EA O'Connor RC Perry VH Tracey I Wessely S Arseneault L. Multidisciplinary research priorities for the COVID-19 pandemic: a call for action for mental health science. Lancet Psychiatry. (2020) 7:547–60. 10.1016/S2215-0366(20)30168-1 32304649 PMC7159850

[10] Kaiser Family Foundation. KFF Health Tracking Poll—July 2020. San Francisco: Kaiser Family Foundation (2020).

[11] Imran N Aamer I Sharif MI Bodla ZH Naveed S. Psychological burden of quarantine in children and adolescents: a rapid systematic review and proposed solutions. Pak J Med Sci. (2020) 36:1106–16. 10.12669/pjms.36.5.3088 32704298 PMC7372688

[12] Saladino V Algeri D Auriemma V. The psychological and social impact of covid-19: new perspectives of well-being. Front Psychol. (2020) 11:577684. 10.3389/fpsyg.2020.577684 33132986 PMC7561673

[13] Danese A Smith P Chitsabesan P Dubicka B. Child and adolescent mental health amidst emergencies and disasters. Br J Psychiatry. (2020) 216:159–62. 10.1192/bjp.2019.244 31718718

[14] Peek L. Children and disasters: understanding vulnerability, developing capacities, and promoting resilience—an introduction. Child, Youth Environ. (2008) 18:1–29. Available online at: http://www.jstor.org/stable/10.7721/chilyoutenvi.18.1.0001 (accessed August 9, 2021).

[15] Orgiles M Morales A Delvecchio E Mazzeschi C Espada JP. Immediate psychological effects of the COVID-19 quarantine in youth from Italy and Spain. Front Psychol. (2020) 11:579038. 10.3389/fpsyg.2020.579038 33240167 PMC7677301

[16] Jiao WY Wang LN Liu J Fang SF Jiao FY Pettoello-Mantovani M. Behavioral and emotional disorders in children during the COVID-19 epidemic. J Pediatr. (2020) 221:264–6. e1. 10.1016/j.jpeds.2020.03.013 32248989 PMC7127630

[17] Christensen D Fahey MT Giallo R Hancock KJ. Longitudinal trajectories of mental health in Australian children aged 4-5 to 14–15 years. PLoS ONE. (2017) 12:e0187974. 10.1371/journal.pone.0187974 29131873 PMC5683648

[18] Bakoula C Kolaitis G Veltsista A Gika A Chrousos GP. Parental stress affects the emotions and behaviour of children up to adolescence: a Greek prospective, longitudinal study. Stress. (2009) 12:486–98. 10.3109/10253890802645041 19206015

[19] Bosquet M Egeland B. The development and maintenance of anxiety symptoms from infancy through adolescence in a longitudinal sample. Dev Psychopathol. (2006) 18:517–50. 10.1017/S0954579406060275 16600066

[20] Bayer JK Ukoumunne OC Lucas N Wake M Scalzo K Nicholson JM. Risk factors for childhood mental health symptoms: national longitudinal study of Australian children. Pediatrics. (2011) 128:e865–79. 10.1542/peds.2011-0491 21890824

[21] Pfefferbaum B Jacobs AK Griffin N Houston JB. Children's disaster reactions: the influence of exposure and personal characteristics. Curr Psychiatry Rep. (2015) 17:56. 10.1007/s11920-015-0598-5 25980513

[22] Pinquart M. Associations of parenting dimensions and styles with externalizing problems of children and adolescents: an updated meta-analysis. Dev Psychol. (2017) 53:873–932. 10.1037/dev0000295 28459276

[23] Sprang G Silman M. Posttraumatic stress disorder in parents and youth after health-related disasters. Disaster Med Public Health Prep. (2013) 7:105–10. 10.1017/dmp.2013.22 24618142

[24] Gibson M Johnson S Field K. The Relationship Between Parent and Child Mental Health: Taking a Family Systems Perspective in Support Services. Melbourne, VIC: FRSA (2019).

[25] Aguilar-Farias N Toledo-Vargas M Miranda-Marquez S Cortinez-O'Ryan A Cristi-Montero C Rodriguez-Rodriguez F. Sociodemographic predictors of changes in physical activity, screen time, and sleep among toddlers and preschoolers in chile during the COVID-19 pandemic. Int J Environ Res Public Health. (2020) 18:176. 10.20944/preprints202012.0038.v1 33383721 PMC7796176

[26] Okely AD Kariippanon KE Guan H Taylor EK Suesse T Cross PL. Global effect of COVID-19 pandemic on physical activity, sedentary behaviour and sleep among 3- to 5-year-old children: a longitudinal study of 14 countries. BMC Public Health. (2021) 21:940. 10.1186/s12889-021-10852-3 34001086 PMC8128084

[27] Guan H Okely AD Aguilar-Farias N Del Pozo Cruz B Draper CE El Hamdouchi A. Promoting healthy movement behaviours among children during the COVID-19 pandemic. Lancet Child Adolesc Health. (2020) 4:416–8. 10.1016/S2352-4642(20)30131-0 32458805 PMC7190292

[28] Carson V Lee EY Hewitt L Jennings C Hunter S Kuzik N. Systematic review of the relationships between physical activity and health indicators in the early years (0–4 years). BMC Public Health. (2017) 17(Suppl 5):854. 10.1186/s12889-017-4860-0 29219090 PMC5753397

[29] Wang H Sekine M Chen X Yamagami T Kagamimori S. Lifestyle at 3 years of age and quality of life (QOL) in first-year junior high school students in Japan: results of the Toyama birth cohort study. Qual Life Res. (2008) 17:257–65. 10.1007/s11136-007-9301-6 18157615

[30] Kuzik N Naylor PJ Spence JC Carson V. Movement behaviours and physical, cognitive, and social-emotional development in preschool-aged children: Cross-sectional associations using compositional analyses. PLoS ONE. (2020) 15:e0237945. 10.1371/journal.pone.0237945 32810172 PMC7433874

[31] Liu W Wu X Huang K Yan S Ma L Cao H. Early childhood screen time as a predictor of emotional and behavioral problems in children at 4 years: a birth cohort study in China. Environ Health Prev Med. (2021) 26:3. 10.1186/s12199-020-00926-w 33413099 PMC7789634

[32] Carson V Ezeugwu VE Tamana SK Chikuma J Lefebvre DL Azad MB. Associations between meeting the Canadian 24-hour movement guidelines for the early years and behavioral and emotional problems among 3-year-olds. J Sci Med Sport. (2019) 22:797–802. 10.1016/j.jsams.2019.01.003 30655179

[33] Chaput JP Gray CE Poitras VJ Carson V Gruber R Birken CS. Systematic review of the relationships between sleep duration and health indicators in the early years (0–4 years). BMC Public Health. (2017) 17(Suppl 5):855. 10.1186/s12889-017-4850-2 29219078 PMC5773910

[34] Harris PA Taylor R Minor BL Elliott V Fernandez M O'Neal L. The REDCap consortium: building an international community of software platform partners. J Biomed Inform. (2019) 95:103208. 10.1016/j.jbi.2019.103208 31078660 PMC7254481

[35] Chorpita BF Moffitt CE Gray J. Psychometric properties of the Revised Child Anxiety and Depression Scale in a clinical sample. Behav Res Ther. (2005) 43:309–22. 10.1016/j.brat.2004.02.004 15680928

[36] Goodman R Ford T Corbin T Meltzer H. Using the Strengths and Difficulties Questionnaire (SDQ) multi-informant algorithm to screen looked-after children for psychiatric disorders. Eur Child Adolesc Psychiatry. (2004) 13 Suppl 2:II25–31. 10.1007/s00787-004-2005-3 15243783

[37] Delisle Nystrom C Alexandrou C Henstrom M Nilsson E Okely AD Wehbe El Masri S. International study of movement behaviors in the early years (SUNRISE): results from SUNRISE Sweden's pilot and COVID-19 study. Int J Environ Res Public Health. (2020) 17:8491. 10.3390/ijerph17228491 33207786 PMC7698175

[38] Draper C Tomaz SA Cook CJ Jugdav SS Ramsammy C Besharati S. Understanding the influence of 24-hour movement behaviours on the health and development of preschool children from low-income South African settings: the SUNRISE pilot study. South Afr J Sports Med. (2020) 32:1–7. 10.17159/2078-516X/2020/v32i1a8415 PMC9924532 36818976

[39] Castro-Schilo L Grimm KJ. Using residualized change versus difference scores for longitudinal research. J Soc Pers Relat. (2017) 35:32–58. 10.1177/0265407517718387

[40] Dalecki M Willits FK. Examining change using regression analysis: Three approaches compared. Sociol Spectr. (1991) 11:127–45. 10.1080/02732173.1991.9981960

[41] Instituto Nacional de Estadísticas—Chile. Censo de Población y Vivienda 2017 Santiago, Chile: Gobierno de Chile (2020). Available online at: https://redatam-ine.ine.cl/redbin/RpWebEngine.exe/Portal?BASE=CENSO_2017&lang=esp (accessed January 12, 2021).

[42] Suor JH Sturge-Apple ML Davies PT Cicchetti D Manning LG. Tracing differential pathways of risk: associations among family adversity, cortisol, and cognitive functioning in childhood. Child Dev. (2015) 86:1142–58. 10.1111/cdev.12376 26081792 PMC4683120

[43] Pechtel P Pizzagalli DA. Effects of early life stress on cognitive and affective function: an integrated review of human literature. Psychopharmacology. (2011) 214:55–70. 10.1007/s00213-010-2009-2 20865251 PMC3050094

[44] Cowie H Myers CA. The impact of the COVID-19 pandemic on the mental health and well-being of children and young people. Child Soc. (2020) 35:62–74. 10.1111/chso.12430 33362362 PMC7753823

[45] Marchetti D Fontanesi L Di Giandomenico S Mazza C Roma P Verrocchio MC. The effect of parent psychological distress on child hyperactivity/inattention during the COVID-19 lockdown: testing the mediation of parent verbal hostility and child emotional symptoms. Front Psychol. (2020) 11:567052. 10.3389/fpsyg.2020.567052 33362632 PMC7758226

[46] Petrocchi S Levante A Bianco F Castelli I Lecciso F. Maternal distress/coping and children's adaptive behaviors during the COVID-19 lockdown: mediation through children's emotional experience. Front Public Health. (2020) 8:587833. 10.3389/fpubh.2020.587833 33330330 PMC7711130

[47] Mental Health Foundation. Coronavirus: Mental Health in the Pandemic. Wave 8: Late November (2020). 2020. Available online at: https://www.mentalhealth.org.uk/our-work/research/coronavirus-mental-health-pandemic/key-statistics-wave-8 (accessed May 1, 2021).

[48] Alcadía, Mayor de Bogotá D,.C. Sistema Distrital de Cuidado Bogotá, Colombia 2020. Available online at: http://www.sistemadecuidado.gov.co/ (accessed January 12, 2021).

[49] Cortinez-O'Ryan A Moran MR Rios AP Anza-Ramirez C Slovic AD. Could severe mobility and park use restrictions during the COVID-19 pandemic aggravate health inequalities? Insights and challenges from Latin America. Cad Saude Publica. (2020) 36:e00185820. 10.1590/0102-311x00185820 33027476

[50] McCormick R. Does access to green space impact the mental well-being of children: a systematic review. J Pediatr Nurs. (2017) 37:3–7. 10.1016/j.pedn.2017.08.027 28882650

[51] Dadvand P Gascon M Markevych I. Green spaces and child health and development. In: Marselle MR Stadler J Korn H Irvine KN Bonn A editors. Biodiversity and Health in the Face of Climate Change. Cham: Springer International Publishing (2019). p. 121–30.

[52] Islam MZ Johnston J Sly PD. Green space and early childhood development: a systematic review. Rev Environ Health. (2020) 35:189–200. 10.1515/reveh-2019-0046 32167931

[53] Engemann K Pedersen CB Arge L Tsirogiannis C Mortensen PB Svenning JC. Residential green space in childhood is associated with lower risk of psychiatric disorders from adolescence into adulthood. Proc Natl Acad Sci USA. (2019) 116:5188–93. 10.1073/pnas.1807504116 30804178 PMC6421415

[54] Aggio D Smith L Fisher A Hamer M. Mothers' perceived proximity to green space is associated with TV viewing time in children: the Growing Up in Scotland study. Prev Med. (2015) 70:46–9. 10.1016/j.ypmed.2014.11.018 25434736 PMC4295935

[55] Pandit L Fauggier GV Gu L Knöll M. How do people use Frankfurt Mainkai riverfront during a road closure experiment? A snapshot of public space usage during the coronavirus lockdown in May 2020. Cities Health. (2020). 10.1080/23748834.2020.1843127. [Epub ahead of print].

[56] Slater SJ Christiana RW Gustat J. Recommendations for keeping parks and green space accessible for mental and physical health during COVID-19 and other pandemics. Prevent Chronic Dis. (2020) 17:E59. 10.5888/pcd17.200204 32644919 PMC7367064

[57] Black MM Walker SP Fernald LCH Andersen CT DiGirolamo AM Lu C. Early childhood development coming of age: science through the life course. Lancet. (2017) 389:77–90. 10.1016/S0140-6736(16)31389-7 27717614 PMC5884058

[58] Shawar YR Shiffman J. Generation of global political priority for early childhood development: the challenges of framing and governance. Lancet. (2017) 389:119–24. 10.1016/S0140-6736(16)31574-4 27717613

[59] Veldman SLC Chin APMJM Altenburg TM. Physical activity and prospective associations with indicators of health and development in children aged <5 years: a systematic review. Int J Behav Nutr Phys Act. (2021) 18:6. 10.1186/s12966-020-01072-w 33413484 PMC7791660

[60] Ministerio de Salud Gobierno de Chile. Recomendaciones de la CMEIS para los comités ético científicos (CECs) en la revisión de protocolos de investigación en contexto de pandemia por COVID-19. 6 junio (2020). In: Comisión Ministerial de Ética en Investigación en Salud, editor. Santiago, Chile.2020.

